# Existence of Prophenoloxidase in Wing Discs: A Source of Plasma Prophenoloxidase in the Silkworm, *Bombyx mori*

**DOI:** 10.1371/journal.pone.0041416

**Published:** 2012-07-25

**Authors:** Yupu Diao, Anrui Lu, Bing Yang, Wenli Hu, Qing Peng, Qing-Zhi Ling, Brenda T. Beerntsen, Kenneth Söderhäll, Erjun Ling

**Affiliations:** 1 Key Laboratory of Insect Developmental and Evolutionary Biology, Institute of Plant Physiology and Ecology, Shanghai Institutes for Biological Sciences, Chinese Academy of Sciences, Shanghai, People’s Republic of China; 2 Department of Applied Biology, Zhejiang Pharmaceutical College, Ningbo, People’s Republic of China; 3 Department of Veterinary Pathobiology, University of Missouri, Columbia, Missouri, United States of America; 4 Department of Comparative Physiology, Uppsala University, Uppsala, Sweden; University of Dayton, United States of America

## Abstract

In insects, hemocytes are considered as the only source of plasma prophenoloxidase (PPO). PPO also exists in the hemocytes of the hematopoietic organ that is connected to the wing disc of *Bombyx mori*. It is unknown whether there are other cells or tissues that can produce PPO and release it into the hemolymph besides circulating hemocytes. In this study, we use the silkworm as a model to explore this possibility. Through tissue staining and biochemical assays, we found that wing discs contain PPO that can be released into the culture medium *in vitro*. An *in situ* assay showed that some cells in the cavity of wing discs have PPO1 and PPO2 mRNA. We conclude that the hematopoietic organ may wrongly release hemocytes into wing discs since they are connected through many tubes as repost in previous paper. In wing discs, the infiltrating hemocytes produce and release PPO probably through cell lysis and the PPO is later transported into hemolymph. Therefore, this might be another source of plasma PPO in the silkworm: some infiltrated hemocytes sourced from the hematopoietic organ release PPO via wing discs.

## Introduction

Prophenoloxidase (PPO) is an important immune protein in insects [1], [2], [3], [4]. It belongs to type-3 copper containing proteins that exist in animals, plants and microbes where they have many different physiological functions [1], [2], [3], [4]. When insects are injected with or naturally infected by microbes or parasites, PPO can be triggered to be converted into its active form phenoloxidase (PO) quickly. For example, bacteria injected into mosquito adults can be melanized within one hour by PO [5]. After melanization occurs, some toxin quinone-like materials are produced to accelerate the killing of invaders [6]. When PPO is activated, it must be cleaved at conserved amino acids by specific serine proteases that are precisely regulated by other proteases, serpins and cofactors like serine protease homologs [2], [4].

Insect PPO is produced by hemocytes in circulation as well as in the hematopoietic organs. Many types of hemocytes have been found to contain PPO [7], [8]. Since insect PPO has no signal peptide, it is still quite unclear how PPO is released from hemocytes into the hemolymph. In the silkworm, the two pairs of wing discs are located in the 2nd and 3rd thoracic segments [9], [10], and the hematopoietic organs are attached on the inside surfaces of wing discs [10], [11]. It is generally thought that PPO cannot be released until hemocytes containing PPO are broken [1]. In insects, hemocytes are produced in hematopoietic organs [12], [13]. In *Drosophila melanogaster*, the hematopoietic organs are called lymph glands that are located along the dorsal vessel [13], [14]. But in Lepidopteran insects (e.g. silkworm), the hematopoietic organs are tightly connected with the wing discs through many tubes [11]. Hemocytes are primarily released during the wandering stage [12]. During the process of hematopoietic organ regeneration after heavy ion beam radiation on the locations of wing discs and hematopoietic organs, the targeted wing discs but not hematopoietic organs became brown, similar to melanization [15]. The exact reasons for this response are still unknown. Since PPO is the main factor that induces *in vivo* melanization, we hypothesized that wing discs may contain or produce PPO, or the plasma PPO attached to the targeted tissue after injury by heavy ion beams, to induce wing disc browning.

In this study we examined whether there is PPO in wing discs. Through tissue staining and biochemical methods, we found that PPO can be transcribed by some cells in the cavity of wing discs. PPO was located in cells in the cavity as well as among cells near the surface of wing discs. When wing discs were cultured *in vitro*, PPO was released into the culture medium. The amount of PPO released by wing discs alone was higher than that by hematopoietic organs. Due to the physical connection of wing discs and hematopoietic organs [11], some hemocytes are probably released by the hematopoietic organs into the wing discs where PPO will be released after cell lysis.

**Figure 1:**
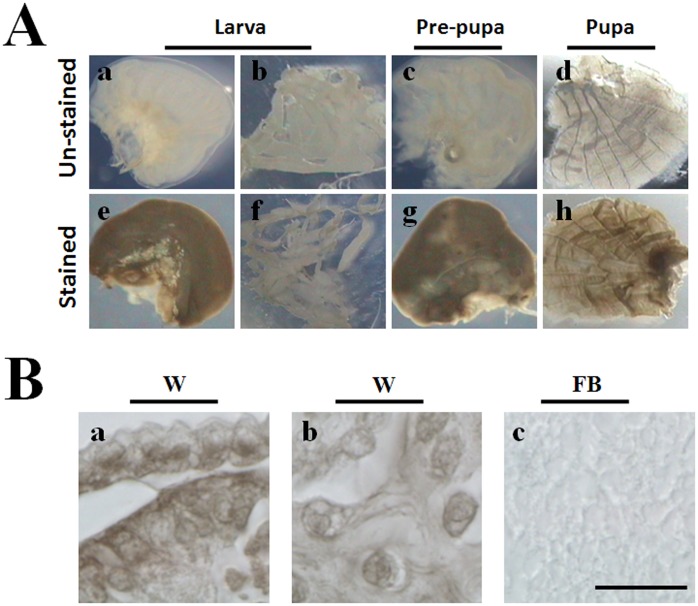
Wing discs are stained black by a mixture of ethanol and dopamine. (A) Wing discs from larvae (e), pre-pupae (g) and pupae (h) could also be stained black by addition of ethanol and dopamine. However, fat bodies (f) were not stained. The unstained wing discs (a, c, d) and fat bodies (b) are white. (B) Microscopic observation of cells in the wing discs after being stained. Cells near the surface of the wing discs (a) and some cells inside the wing discs (b) were stained black. Fat body cells could not be stained (c). W: wing discs; FB: fat bodies. Bar: 10 µm.

## Results

### Existence of Prophenoloxidase (PPO) in Wing Discs

The heavy ion beam targeted wing discs of *Bombyx mori* became brown *in vivo* [15], indicating that the wing discs might have PPO. We used a mixture of ethanol (PPO activator) and dopamine (PPO substrate) to easily identify PPO-positive hemocytes [7], [8]. Using this mixture, we stained wing discs dissected from larvae on day 3 of the fifth larval stage (V-3) (Fig. 1A–e), pre-pupae (Fig. 1A–g) and pupae (Fig. 1A–h). The results show that all wing discs became melanized compared to wing discs without staining (Fig. 1A-a,c,d). After staining, many cells on the side of wing discs (Fig. 1B-a) and inside the wing discs (Fig. 1B-b) were melanized. However, fat body cells were not melanized after staining (Fig. 1A–f, 1B–c), which are similar to those without staining (Fig. 1A–b). When phenylthiourea (PTU), a PPO strong inhibitor [16], was added to the staining mixture, wing discs could not be stained black (data not shown). These results indicate that there might be PPO in the wing discs.

**Figure 2:**
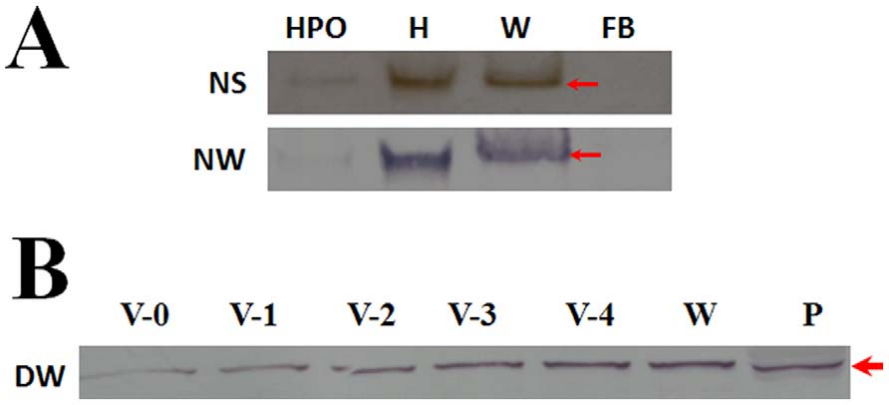
Wing discs contain PPO. (A) Cell lysates from hematopoietic organ (HPO), hemocytes (H), wing discs (W) and fat bodies (FB) were separated by native gel for PPO activity detection (top panel) and Western blot detection by antibody against silkworm PPO (bottom panel). (B) PPO in wing discs at different developmental stages. Plasma (P) (0.1 µl) was loaded as the positive control. Equal numbers of anterior and posterior wing discs were sonicated. For each lane, approximately 15 µg proteins were loaded. PPO was detected in wing discs at the different developmental stages. NS: Native gel separation for PPO Staining; NW: Native gel separation for the followed Western blot detection; DW: Denatured protein for Western blot.

**Figure 3:**
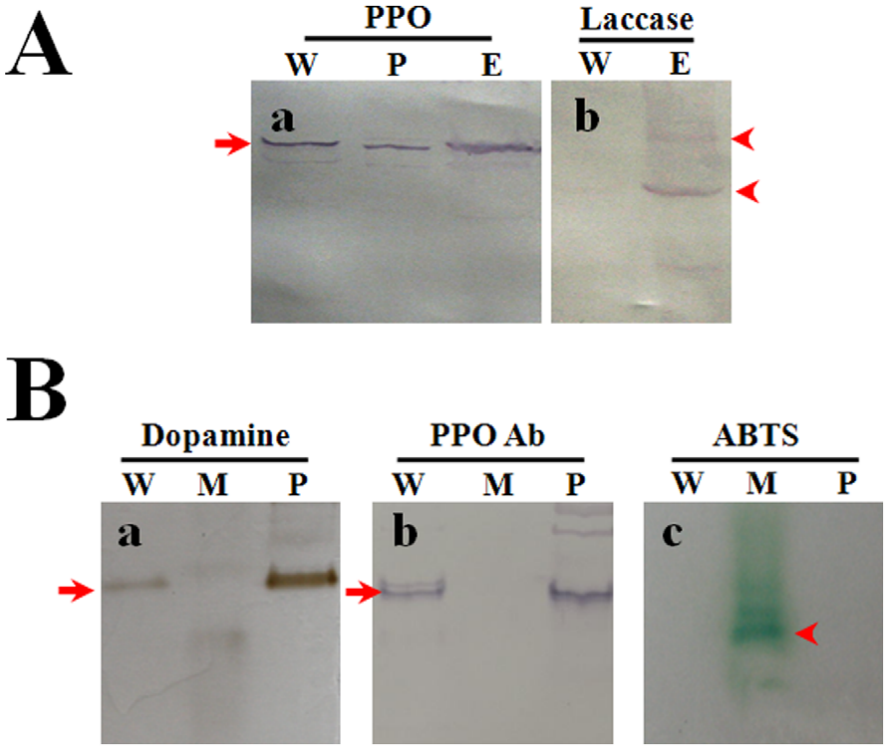
Wing discs do not exhibit any laccase activity. Laccase is one of the enzymes in insects that may also oxidize phenol to produce melanin materials. Cell lysates of wing discs (W) and epidermis (E) from larvae on V-3 and cell lysates of mushroom (M) and plasma (P) were assayed by Western blot and native gel staining for laccase detection. For each lane, approximately 15 µg proteins were loaded. (A) No laccase was detected in wing discs using a denatured Western blot. PPO in the lysates was detected with a primary antibody against silkworm PPO. Laccase, however, was detected in epidermis as the arrowhead indicates. (B) No active laccase in the wing discs. Laccase activity was detected in the mushroom lysate that served as a positive control. After native gel separation, the gel was stained to show PPO activity (a) by incubating it with dopamine or to show laccase activity by incubating it with ABTS (c) after being activated by ethanol. One native gel was further assayed by Western blot to detect PPO protein (b). The arrows indicate PPO in wing discs and plasma. The arrowhead points to active laccase in the mushroom lysate. No active laccase in the wing discs or plasma was observed. W: wing discs; P: plasma; E: epidermis; M: mushroom.

**Figure 4:**
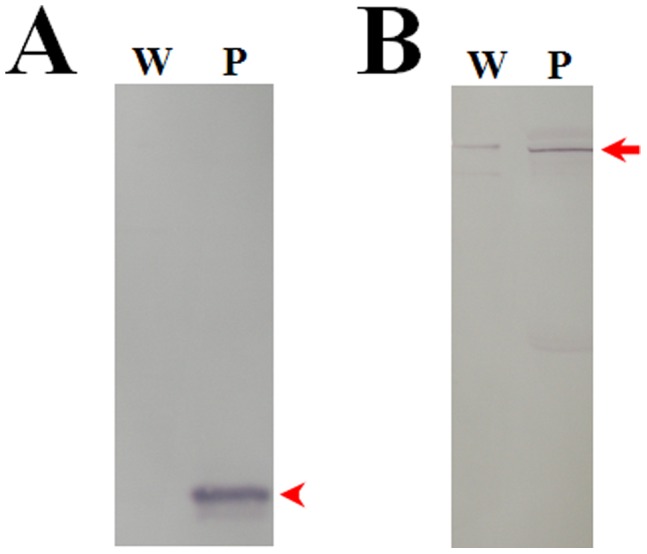
PPO in the wing discs is not due to plasma contamination. Silkworm larvae were injected with formalin-killed *Escherichia coli* for 12 h. Wing discs and plasma were sampled from the bacteria-injected larvae. For each lane, approximately 15 µg wing disc protein or 0.1 µl plasma was loaded. The samples were detected with silkworm lysozyme antibody (A) and PPO antibody (B) by Western blot. In plasma but not in the wing discs, there was lysozyme as indicated by the arrowhead (A). PPO was detected in both plasma and wing discs as indicated by the arrow (B). There was no direct physical connection between wing discs and hemolymph.

Lysates were made from the separated wing discs, hematopoietic organs, hemocytes and fat bodies that were dissected from larvae on V-3 and the supernatants were assessed via native gel analysis. The results show that there is a band at the same position as PPO from the hematopoietic organs and hemocytes after native gel analysis of PPO activity (Fig. 2A-Up panel). A Western blot was done after native gel separation and the corresponding positively-stained band was detected by antibody against silkworm PPO (Fig. 2A-Bottom panel). No band was detected in fat bodies by native gel staining or Western blot. A time-course assay was performed to assess PPO change in wing discs during the whole fifth larval stage by Western blot. The amount of PPO in wing discs increased with time (Fig. 2B). To compare the anterior and posterior wing discs, the amounts of PPO were almost the same (data not shown). The corresponding band that had PO activity on the native gel was excised for a LC/MS-MS assay and peptide fragments of PPO were detected (Fig. S1) and it was confirmed that this band contains PPO1 and PPO2. In insects, two additional enzymes, peroxidase and laccase, may also oxidize phenols to produce melanin [2]. No peroxidase activity was detected in wing discs (data not shown). After a denaturing gel (SDS-PAGE) separation, antibody against *Manduca sexta* laccase was used to determine if there is laccase in wing disc and epidermis from larvae on V-3. The results show that only epidermis has laccase (Fig. 3A right panel). However, just like in plasma, PPO was detected not only in wing discs but also in epidermis (Fig. 3A left panel). To further prove if there is active laccase in larval wing discs, samples of plasma, wing disc and mushroom lysates were run on native gel for PPO and laccase activities detection. The results show that there was a PPO band in wing disc and plasma, but not in mushroom (Fig. 3B-a), which was also proved by Western blot after native gel separation (Fig. 3B-b). Laccase activity was detected in the mushroom sample but not with wing discs or plasma (Fig. 3B–c). Consequently, there is no laccase in larval wing discs.

**Figure 5:**
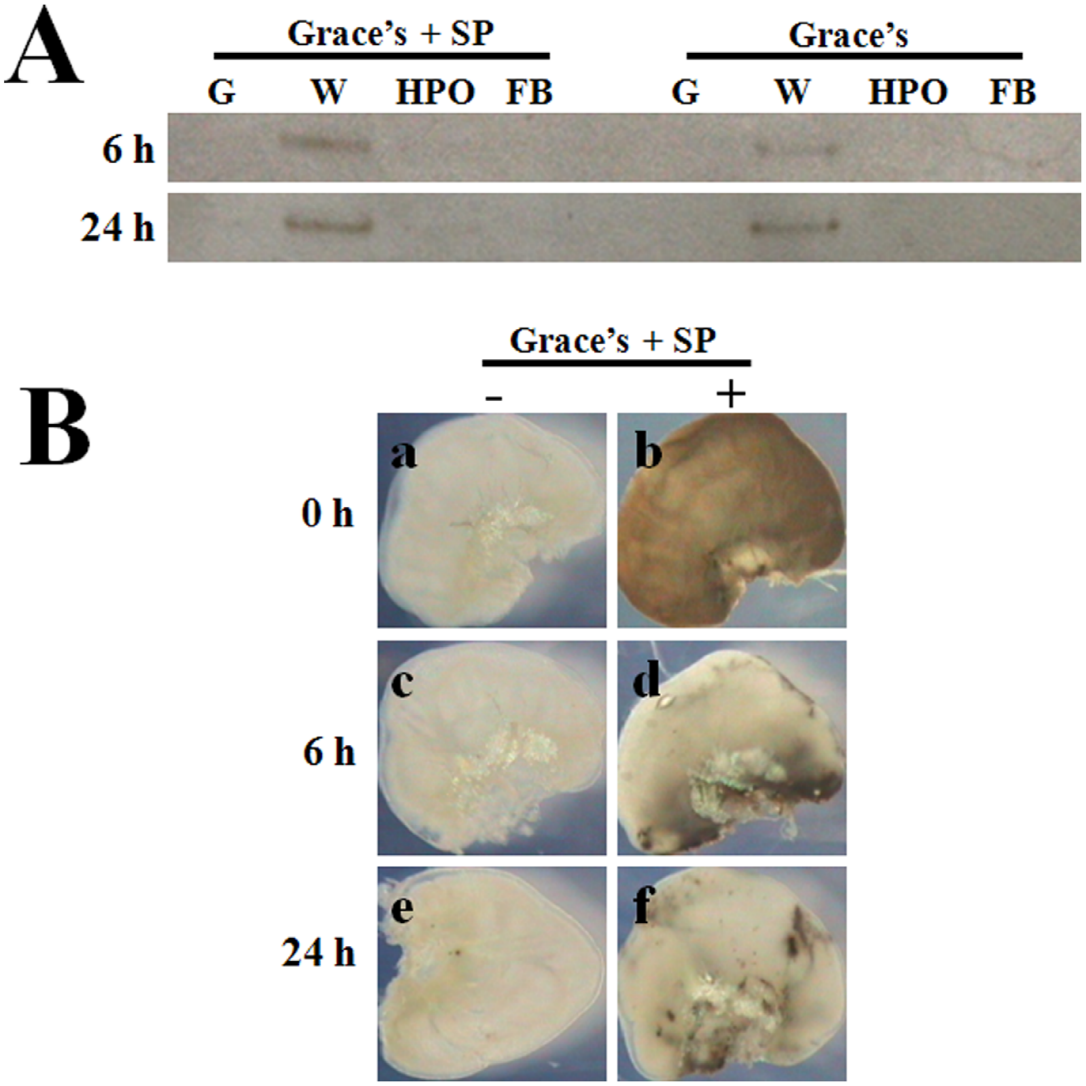
PPO is released from cultured wing discs. The separated wing discs (W), hematopoietic organ (HPO) and fat bodies (FB) were cultured in Grace’s medium with or without Silkworm Plasma (SP). (A) Greater amounts of PPO were released from wing discs than from hematopoietic organs on average at 6 and 24 h post culture. No PPO was released from fat bodies, and there was also no PPO in fresh Grace’s culture medium (G). (B) Cultured wing discs were stained as shown in Fig. 1 at 0, 6, 24 h post culture. To compare with the ones without staining (a), the stained (b) wing discs became melanized at the initiation of culture (0 h). When wing discs were cultured for 6 and 24 h, only the area where the wing discs and hematopoietic organs are connected was stained black (d, f) if compared with those without staining (c, e), suggesting the release of PPO from wing discs.

Since wing discs are immersed in hemolymph *in vivo,* it is possible to contaminate wing discs with hemocytes or plasma PPO. Larvae on V-3 were injected with killed *Escherichia coli* for 12 h. Lysozyme containing a signal peptide is secreted into plasma. The protein was not found in the hematopoietic organs (data not shown). Therefore, lysozyme is suitable as a probe to show whether there are large molecules communication between the wing discs and hemolymph. According to the Western blot assay using PPO and lysozyme antibodies separately as the primary antibody, lysozyme was detected in plasma alone but not in wing discs (Fig. 4A). However PPO was found in both wing discs and plasma (Fig. 4B). The results indicate that there are no physical holes between wing discs and hemolymph; otherwise, lysozyme should be detected in wing discs.

**Figure 6:**
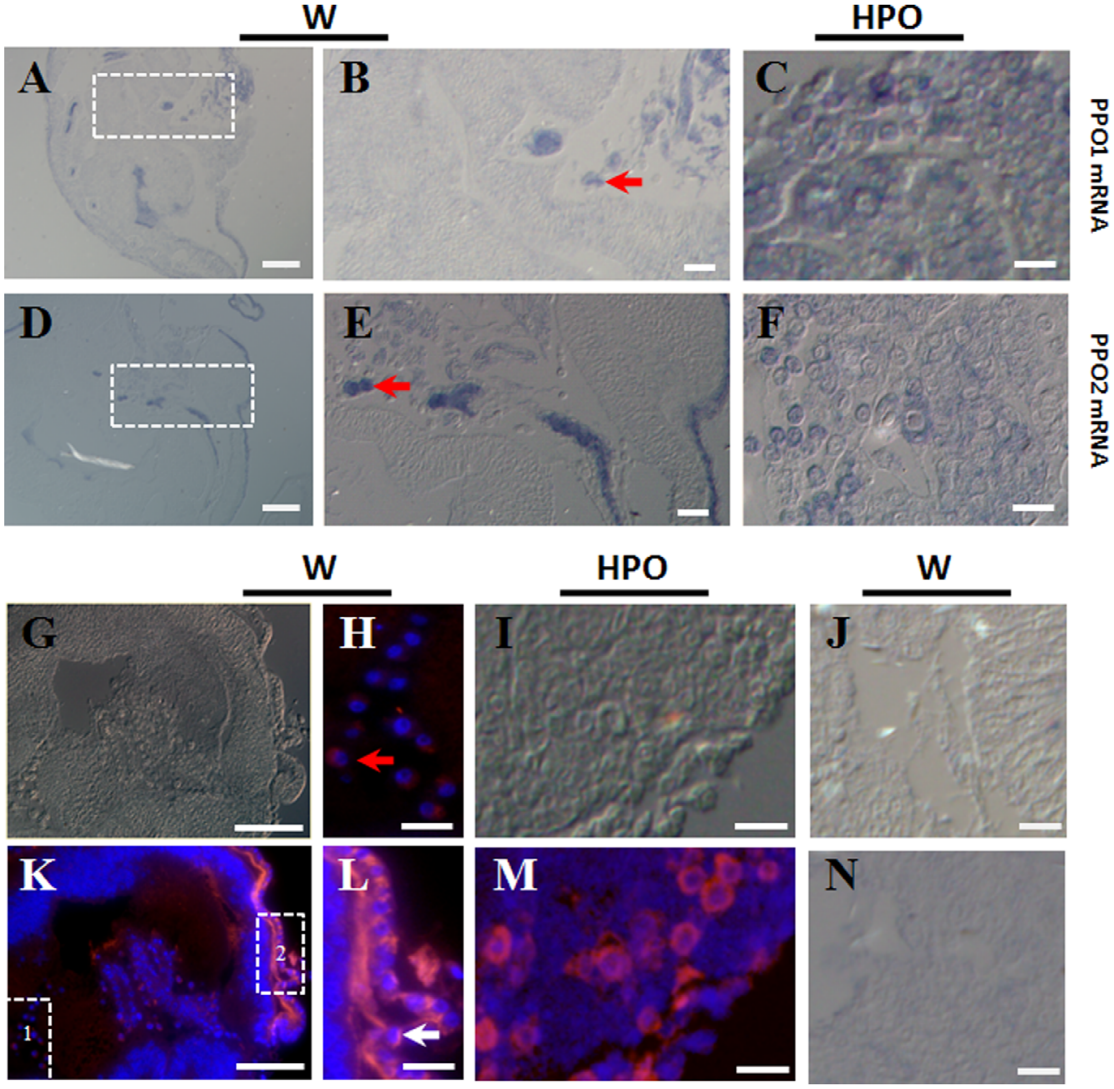
Location of PPO protein and PPO mRNA in wing discs. Wing discs (W) and hematopoietic organs (HPO) of larvae on V-3 were fixed and sectioned for PPO immuno-staining and PPO mRNA detection in the same condition. (A–F) Detection of PPO1 (A–C) and PPO2 (D–F) mRNA in the wing discs (A, B, D, E) and hematopoietic organs (C, F). In the hematopoietic organs, there are many cells showing PPO1 and PPO2 mRNA signals (C, F).Wing discs imaged at low magnification were shown (A, D). The framed areas in (A) and (D) were shown in (B) and (E) respectively at high magnification. The arrow-indicated several cells accumulated together in the cavity of wing discs had PPO mRNA signals (B, E). (J and N) Negative control using sense mRNA probes. (G–I, K–M) Detection of PPO proteins in cells of wing disc (G, H, K, L) and hematopoietic organs (I, M). Cells with red fluorescence in wing discs and hematopoietic organs have PPO proteins. The enlarged picture in (H) was from the dot-lined frame 1 in (K). A few cells in the cavity of wing discs (H) were found to have PPO. Among cells on the brisk of the wing discs, the PPO signal was also strong (L), which is shown in the dot-lined frame 2 in (K). DAPI (blue fluorescence) was used for nuclei counterstaining. The arrows point to some cells containing PPO proteins. Bar: (A, D, G, K) 80 µm; (B, E) 50 µm; All others: 20 µm.

### PPO Released from the Cultured Wing Discs *in vitro*

The separated wing discs, hematopoietic organs and fat body from the same larvae on V-3 were cultured separately in Grace’s medium with or without 10% silkworm plasma heated at 60°C for 30 min to inactivate plasma PPO [11], [17]. The native gel assays show that at 6 and 24 h post culture much more PPO was released from wing discs than from hematopoietic organs (Fig. 5). No PPO was released from fat bodies, indicating that there was no contamination by hemocytes or plasma PPO during the process of preparation. There was no PPO in Grace’s medium containing the heated silkworm plasma alone.

**Figure 7:**
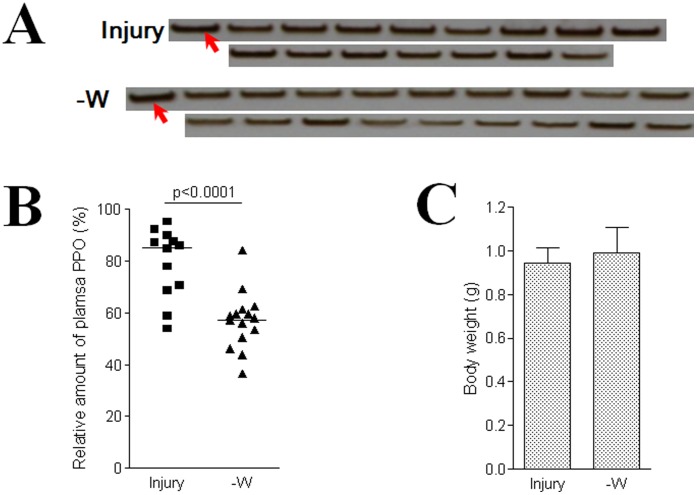
A surgical operation to remove wing discs and hematopoietic organs decreased plasma PPO. (A) Individual plasma PPO (0.5 µl) was separated and detected by a native gel assay and PPO bands were normalized with the standard plasma (0.55 µl) from naïve larvae as indicated by the arrows. Silkworm larvae on V-0 were surgically operated on to remove the wing discs along with the hematopoietic organs (-W), and a sham-operation was performed underneath the wing discs (Injury). Each lane stands for an individual larva. (B) The relative amounts of plasma PPO were calculated. When wing discs with hematopoietic organ were removed, the plasma PPO was significantly lower as compared to the sham operated larvae (Injury). Each dot corresponds to the relative amount of PPO in one silkworm larva. The average for each group is indicated by a horizontal black bar (n = 15). Significant differences were calculated using an unpaired *t*-test. (C) There were no obvious differences in body weight between those silkworm larvae with wing discs removed or those receiving the sham-operation.

The cultured wing discs were also removed for tissue staining at the same time. Before the initiation of the culture, the wing discs could be stained black (Fig. 5B-b). At 6 and 24 h post culture, the wing disc was stained black at the place where it is connected with the hematopoietic organ (Fig. 5B–d, 5B–f). Without staining, the wing discs did not turn black at each time-point assayed (Fig. 5B-a, c, e). Thus, it appears that PPO was released from the wing discs during the process of culturing.

### PPO Transcription and Expression by Cells in the Wing Discs

*In situ* assay (Fig. 6A–6F) and immuno-staining (Fig. 6G–6I and 6K–6M) also indicate that there are PPO mRNA and PPO proteins in cells of hematopoietic organs and wing discs. Cells in the hematopoietic organs (Fig. 6C, 6F, 6M) serves as positive controls for observing the corresponding signals in cells of wing discs (Fig. 6B, 6E, 6H, 6K, 6L). A few cells accumulated in the cavity of the wing disc had PPO1 (Fig. 6B) and PPO2 (Fig. 6E) mRNA signals. Those cells could not be detected by sense mRNA probes (Fig. 6J and 6N). The immuno-staining results show that many cells in the cavity of wing discs had PPO (Fig. 6H). There was also PPO in cells on the brisk of the wing discs (Fig. 6L). However, the RT-PCR assays show that the mRNA level of PPO1 or PPO2 was not significantly higher than that from the hematopoietic organs and the hemocytes (Fig. S2). Since wing discs have a physical connection with the hematopoietic organ through many tubes [11], we conclude that those cells with positive signal PPO mRNA were hemocytes originating from the hematopoietic organs. The infiltrated hemocytes will release PPO that can bind to the cells of wing disc since PPO has a sticky property [1], by which to get many cells in the wing disc positively immuno-stained.

**Figure 8:**
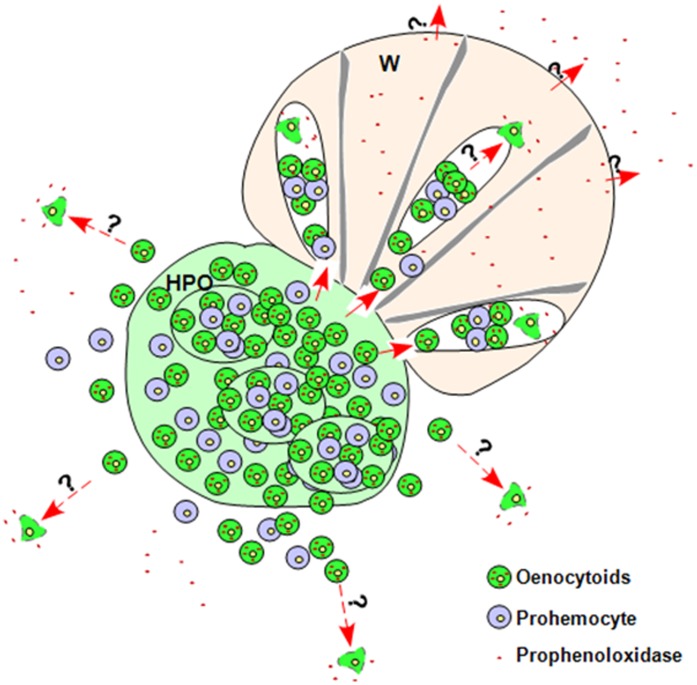
Model of silkworm prophenoloxidase (PPO) releasing. In a typical Lepidoptera insect such as *Bombyx mori*, the wing disc (W) connects with the hematopoietic organ (HPO) through many tube-like structures [11]. In the hematopoietic organs, there are many cysts containing mainly prohemocytes and oenocytoids [31]. Due to the physical connection between wing discs and hematopoietic organs, hemocytes may enter the wing disc and accumulate in the cavity. Prohemocytes and oenocytoids can be released into circulation where oenocytoids then will release PPO through cell lysis via an unclear mechanism. Finally, PPO in the wing disc will be released into hemolymph based on the *in vitro* assay (Fig. 5). The interrogation marks (?) indicate progresses that are still unclear.

### Contribution of Wing Discs to Hemolymph PPO

Silkworm larvae on the first day of the fifth larval stage (V-0) were surgically operated to remove two pairs of wing discs along with hematopoietic organs after feeding for 6 h as previously described [18]. The sham operated control was performed by making four incisions under the wing discs. Preliminary data show that on each day after the operation there were no differences in plasma PPO among the sham-operation and naïve larvae; however, plasma PPO in larvae with the wing discs removed was lower than in larvae that received the sham-operation (data not shown). We choose to assess plasma PPO levels four days after surgically removing all the wing discs when the naïve larvae were almost about to enter the wandering stage and hemocytes should be released in large numbers [12]. Individual plasma PPO was separated on a native gel using the same volume of naïve plasma as a standard (Fig. 7A). PPO-positive bands were normalized with the standard (as 100%) and the PPO amounts were calculated. After removing all four wing discs along with the hematopoietic organs, the plasma PPO decreased significantly (Fig. 7B). However, the operation had no effect on larval growth (Fig. 7C). Since most PPO-positive hemocytes in the hematopoietic organs will also be released into hemolymph, the exact amount of PPO released via wing discs is still difficult to account.

## Discussion

In insects, PPO produced by hemocytes is released into the hemolymph via a very unclear mechanism [1]. Besides circulating hemocytes, as well as hemocytes in the hematopoietic organ, it is unknown whether there are other tissues or cells that can also produce PPO. While cuticle was found to have PPO, it seems that transcription of PPO does not occur in the cuticle tissues [1]. However, the hindgut cells of *Bombyx mori* was recently found to produce PPO and that this PPO was released into the hindgut content to induce the melanization of feces and reduce bacteria number in the hindgut [19]. *In vivo* insect PPO is activated through a proteolytic cascade terminating with a serine proteinase cleaving PPO to produce the active enzyme PO. However, *in vitro* PPO can also be activated by many chemicals like SDS, ethanol and propanol by a hitherto unknown mechanism [1]. Through ethanol activation and dopamine staining, we found that larval wing discs were stained black (Fig. 1), which indicates that there might be PPO present. Peroxidase and laccase are two enzymes that might also oxidize monophenol and diphenol substrates to produce melanin [2]. Our enzyme activity assay results using native gels and Western blots show that there is no laccase in wing discs. And no peroxidase activity was detected (data not shown). Through native gel staining and Western blot assays (Fig. 2), we found that wing discs have PPO whose identity was further confirmed by mass spectrometry (LC–MS/MS) (Fig. S1). When we prepared for this paper, PPO was also found in the hindwing of *Tribolium castaneum* through proteomics assay, and the change of PPO transcription was also found higher in the elytra than in the hindwing [20]. When pro-phenoloxidase III was knocked-down in *Armigeres subalbatus*, the adult wings became malformed [21]. These findings indicate the existence of PPO in the wing discs or wings and that PPO may be there to control the wing disc development but this proposal needs further detailed studies.

When the wing discs were cultured *in vitro*, PPO in the wing discs was obviously released as shown by the native gel assay (Fig. 5A) and tissue staining (Fig. 5B). Although we did not observe PPO1 or PPO2 mRNA in cells near the surface of the wing discs, we found that among these cells the protein signals are strong (Fig. 6L). These results suggest that PPO is continuously transferred to the brisk of wing disc for release. Consequently, the accumulation of PPO on the brisk makes the signal strong among these cells since PPO has sticky property [1]. In the silkworm, the wing disc and hematopoietic organ are not separate tissues because they have a number of tube-like tissues that connect with each other [11]. This means that hemocytes may be released from the hematopoietic organs and enter the wing discs. Cells with PPO1 and PPO2 mRNA signals are primarily located in the wing disc cavity (Fig. 6B, 6E). When we performed RT-PCR to compare PPO1 and PPO2 transcription in the wing discs and hematopoietic organs, the amplified PPO fragments were not higher from wing disc cDNA than from hematopoietic organ cDNA (Fig. S2). Therefore, PPO transcription occurred in only a few cells. The only reasonable explanation is that the hematopoietic organ had already released some hemocytes into the wing discs. Unfortunately, we have no specific protein makers to identify hemocytes from cells of the wing discs in the silkworm. In the wing discs, the invading hemocytes will release PPO into the cavity through cell lysis. Finally, PPO will leave the wing discs and enter the hemolymph in unknown mechanism. As illustrated in Fig. 8, in addition to the release of PPO from circulating hemocytes into the hemolymph, the hematopoietic organ may also release PPO via the attached wing disc.

## Materials and Methods

### Insect Feeding and Dissection

*Bombyx mori* larvae (Nistari) were reared on mulberry leaves at 25°C under a 12-h photoperiod. Larval age was reported in days as described by Kiguchi et al. [22]. Briefly, it is noted as day 0 after 5th larval ecdysis (V-0), and the 2nd as V-1 and so on. Most 5th instar larvae began to wander on day 6, which is noted as the wandering stage (W). To obtain samples for Western blot, immuno-staining, or native gel assay, silkworm larvae at desired stage during the fifth stage and wandering stage were dissected in autoclaved 0.85% NaCl solution after bleeding. The dissected tissues were washed in fresh 0.85% NaCl solution three times to remove hemolymph. Larvae were also bled for hemolymph that was transferred to a new tube for centrifuging at 5,000 × g for 3 min. The supernatant (plasma) was stored at −80°C for further use.

### Surgical Extirpation of Wing Discs

Silkworm hematopoietic organ can be extirpated with wing discs without affecting larval growth and development [18]. In the same way, two pairs of wing discs of larvae on the first day of the fifth larval stage were surgically removed as described [18]. A sham operation was performed by tearing the cuticle near the four wing discs. After the operation, the silkworm larvae were fed as normal. Four days later, plasma was sampled from each larva with wing discs removed or from each naïve or sham-operated larva.

### Wing Disc Staining

The dissected or cultured wing discs were incubated in 10 mM dopamine containing 30% ethanol for staining at room temperature for 5 min. Dopamine is a PPO substrate and ethanol can be used to activate PPO [1], [7]. Phenylthiourea (PTU), a PPO inhibitor [16], was also added to assess that the observed staining with dopamine is due to PO activity.

### Tissue Culture and Native Gel Assay

Wing discs, hematopoietic organs, and fat bodies dissected from 4 larvae were separately cultured in 500 µl Grace medium containing 10% heated silkworm plasma in which PPO was inactivated at 60°C for 30 min after modifying the previously published method [11], [17]. The culture medium (50 µl) was sampled at scheduled times to determine plasma PPO activity by native gel assay. For each lane, 12 µl (sample medium) was loaded. For cell lysates from the wing discs (for PPO detection) and mushroom (for laccase detection), approximately 15 µg was loaded for each lane. PO activity in the native gel was performed as described previously [8], and laccase activity in mushroom was detected by incubation in 10 mM Tris buffer (pH 7.4) containing 2 mM 2,2′-azino-bis(3-ethylbenzthiazoline-6-sulfonic acid (ABTS) [23].

### Immune Challenge and Lysozyme Detection

V-3 silkworm larvae were injected with 5×10^6^ formalin-killed *Escherichia coli* (*E. coli*) cells suspended in sterilized 0.85% NaCl solution or 0.85% NaCl alone for immune challenge for 12 h [19]. The silkworm larvae then were bled for plasma. Wing discs were also sampled as described above. These samples were used for detecting lysozyme by Western blot.

### Immuno-histochemistry

Wing discs were dissected and fixed overnight at 4°C in Bouin’s fluid [24]. Samples were sectioned, deparaffinized, and immuno-stained. Antibody against the silkworm PPO (1∶1,000) was used as the first antibody (a gift from Dr. T. Asano) [25], and rhodamine-conjugated goat anti-rabbit IgG (1∶200) was used as the second antibody. DAPI was used to counter-stain nuclei before the last washing for 5 min. All pictures were taken using a fluorescent microscope (Olympus BX51) with differential interference contrast using the appropriate filter.

### *In situ* Hybridization

*Bombyx mori* PPO1 and PPO2 mRNA were detected in wing discs and hematopoietic organs by *in situ* hybridization following described methods [24], [26]. The antisense and sense RNA probe was labeled with digoxigenin by *in vitro* transcription with T7 RNA polymerase in a reaction containing digoxigenin-UTP (DIG RNA Labeling Kit, Germany). The primers for cloning PPO1 and PPO2 and those for synthesizing antisense and sense RNA probes are listed in Table S1.

### LC-MS/MS

Cell lysate from wing discs was separated by native gel. One gel was used for Western blot assay (see below) to show the position of PPO. Another gel was stained by Coomassie Brilliant Blue R250 (CBB) and the band at the same position with PPO detected was excised for liquid chromatography tandem mass spectrometry (LC–MS/MS) analysis as previously described [19].

### Semiquantitative RT-PCR

Semiquantitative RT-PCR was used to examine relative abundance of selected genes in different tissues. Total RNA of each tissue was isolated using Trizol, and the first-strand cDNA was synthesized as described above. The primers are listed in Table S1. Ribosomal protein 3 (rps3) was used as an internal control for equal RNA loading [27]. PCR products were separated via 1% agarose gel electrophoresis.

### SDS-PAGE and Western Blot Analysis

Approximately 15 µg protein was loaded per lane unless otherwise mentioned, and SDS-PAGE and Western blot assay were performed as previously described [28]. Antibody against the silkworm PPO (1∶5,000), or lysozyme (a gift from Dr. K. Suzuki; 1∶5,000) [29], or *Manduca sexta* laccase (a gift from Dr. M. Kanost; 1∶2,000) [30] was used as the primary antibody, and the AP-conjugated goat anti-rabbit IgG (1∶5,000) was used as the second antibody.

## Supporting Information

Figure S1 **Identification of the proteins in the bands that exhibit prophenoloxidase (PPO) activity.** (A) PPO in the cell lysate from wing discs was detected by western blot (lane 1) using the antibody against PPO Another lane (lane 2) was stained by Coomassie Brilliant Blue R250 (CBB) to target the band containing PPO for liquid chromatography tandem mass spectrometry (LC–MS/MS) analysis. The arrowheads indicate the position of PPO. (B, C) Peptides were identified as PPO1 (B) and PPO2 (C) as indicated. Other proteins were also identified and these results are not shown.
(TIF)

Figure S2 **Semi-quantitative RT-PCR analysis of transcription of PPO1 and PPO2 in hematopoietic organs (HPO), hemocytes (H), wing discs (W) and fat bodies (FB).** The number of PCR cycles used was 35. Ribosomal protein 3 (rps3) was used as an internal control for equal RNA loading.
(TIF)

Table S1 **Primers for RT-PCR analysis and** ***in situ*** **hybridization.**
(XLS)
