# Supplementary figures and images for: Existence of Prophenoloxidase in Wing Discs: A Source of Plasma Prophenoloxidase in the Silkworm, Bombyx mori
Source: PLoS One. 2012 Jul 25;7(7):e41416. doi: 10.1371/journal.pone.0041416 (PMC3405132; doi:10.1371/journal.pone.0041416)

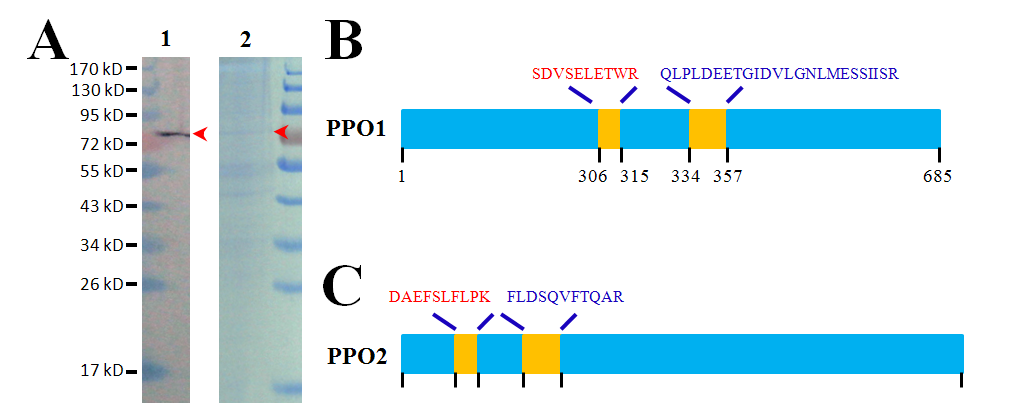

Supplement: Figure S1 — Identification of the proteins in the bands that exhibit prophenoloxidase (PPO) activity. (A) PPO in the cell lysate from wing discs was detected by western blot (lane 1) using the antibody against PPO Another lane (lane 2) was stained by Coomassie Brilliant Blue R250 (CBB) to target the band containing PPO for liquid chromatography tandem mass spectrometry (LC–MS/MS) analysis. The arrowheads indicate the position of PPO. (B, C) Peptides were identified as PPO1 (B) and PPO2 (C) as indicated. Other proteins were also identified and these results are not shown. (TIF) [file pone.0041416.s001.tif]

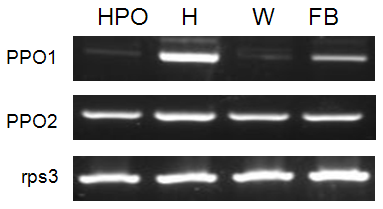

Supplement: Figure S2 — Semi-quantitative RT-PCR analysis of transcription of PPO1 and PPO2 in hematopoietic organs (HPO), hemocytes (H), wing discs (W) and fat bodies (FB). The number of PCR cycles used was 35. Ribosomal protein 3 (rps3) was used as an internal control for equal RNA loading. (TIF) [file pone.0041416.s002.tif]
